# Effect of longer femoral head on leg length, offset, and range of motion in total hip arthroplasty: a simulation study

**DOI:** 10.1038/s41598-024-52264-4

**Published:** 2024-01-21

**Authors:** Tomohiro Shimizu, Takuji Miyazaki, Shunichi Yokota, Hotaka Ishizu, Daisuke Takahashi, Norimasa Iwasaki

**Affiliations:** Department of Orthopaedic Surgery, Faculty of Medicine and Graduate School of Medicine, Hokkaido University, Kita-15, Nish-7, Kita-ku, Sapporo, 060-8638 Japan

**Keywords:** Medical research, Medical imaging

## Abstract

In this study, we investigated the relationship between head length, leg length, offset, and dislocation resistance using range of motion (ROM) simulations based on computed tomography data to examine if a longer femoral head reduces the risk of dislocation. The femoral components were set to eliminate leg length differences with a + 0 mm head, and variations for + 4-, + 7-, and + 8-mm heads were analyzed. Offset and ROM were assessed when longer heads were used, with the leg length adjusted to be similar to that of the contralateral side. While internal rotation at flexion and external rotation at extension increased with + 4-mm longer heads, the + 7- and + 8-mm heads did not increase dislocation resistance. When adjusting for leg length, the longer heads showed no significant differences in offset and ROM. Enhancing dislocation resistance by solely increasing the offset with a longer head, while simultaneously adjusting the depth of stem insertion, may be a beneficial intraoperative technique. Although a + 4-mm longer head possibly increases ROM without impingement, heads extended by + 7 or + 8 mm may not exhibit the same advantage. Therefore, surgeons should consider this technique based on the implant design.

## Introduction

Dislocation is a serious complication of total hip arthroplasty (THA) and has been reported in 0.5–10% of primary THAs^1^; it is the number one indication for revision hip arthroplasty^2^. The surgical techniques to avoid the risk of dislocation are proper placement of the acetabular implant^3^, selection of a stem with an appropriate offset^4^, and insertion of the stem at an appropriate anteversion angle^5^. One cause of decreased patient satisfaction with THA is the postoperative leg length difference (LLD)^6^. Because a postoperative LLD exceeding 10 mm may decrease patient satisfaction^7–9^, clinicians are required to minimize the LLD.

To achieve reconstruction similar to that of the normal hip joint and obtain dislocation resistance, understanding the anatomy and selecting an appropriate implant design are important. In addition to the surgical approach^10^ and implant positioning^3^, the intraoperative approach to achieve these goals is using a longer femoral head and extended offset polyethylene liner^11^. Because the offset extended liner is reported to have a relatively higher failure rate^11^, a longer femoral head may be preferentially used. Although few reports address the long-term effects of design changes on range of motion (ROM)^12–14^, little information is available regarding the actual amount of leg extension and offset using a longer head.

Recent meta-analyses have emphasized the effectiveness of cemented stems in older patients^15^, renewing interest in this technique^16^. This has increased the application of cemented stems in certain countries^17^. Cemented stems offer advantages such as adjusting depths and insertion angles, and longer heads facilitate intraoperative leg length and offset modifications. Although these factors are crucial during THA, research on the specific impacts of cemented stems and longer heads on offset, leg length, and impingement has been lacking. Hence, in this study, we aimed to investigate the relationship between head length, leg length, offset, and ROM in three dimensions using a simulation study based on computed tomography (CT) data to clarify whether a longer femoral head reduces the risk of dislocation.

## Materials and methods

### Ethics statement

Our study was performed in accordance with the relevant guidelines of Hokkaido University Hospital and was approved by its Research Ethics Review Committee. Our research protocol for human samples used in this study was approved by the Research Ethics Review Committee of Hokkaido University Hospital (Approval ID: 019-0031). Informed consent for using samples in our research was obtained from all participants.

### Participants and data collection

Seventeen participants (9 men and 8 women) with osteonecrosis of the femoral head (ONFH) who underwent unilateral trans-trochanteric curved varus osteotomy at our institution between 2016 and 2021, provided informed consent to participate in this study. The average age of the participants was 32.1 years (range, 18–45 years), height 165.2 cm (147.2–175.3 cm), and weight 66.3 kg (45.5–99.7 kg). Preoperative CT images of the patients were used for this study. These patients with ONFH were chosen because their anatomical features closely resemble those of healthy individuals, without acetabular dysplasia, osteophytes, or leg length discrepancies commonly found in osteoarthritis. We accessed data that could identify individual participants during or after data collection. Table 1 summarizes the radiographic parameters and implant sizes used in this study. This study had no cases of developmental dysplasia of the hip (DDH) and hip osteoarthritis (OA). According to the Association Research Circulation Osseous (ARCO) classification^18^, 5 stage II and 12 stage IIIa cases were present.

**Table 1:** Radiographic parameters and implant size.

	Mean (range)	
Center of edge, degree	34.1 (26.0–41.1)	
Sharp angle, degree	39.8 (35.2–44.1)	
Acetabular head index, %	82.5 (77.1–88.8)	
ARCO classification		
Stage II, hip	5	
Stage IIIA, hip	12	

Cup size	Trident cup	GS cup
48 mm cup, hip	4	
50 mm cup, hip	3	
52 mm cup, hip	5	
54 mm cup, hip	4	
56 mm cup, hip	1	

Stem size	Exeter stem	VLIAN stem
37.5-0, hip	7	
37.5-1, hip	8	
37.5-2, hip	1	
37.5-3 hip	1	
40-1, hip		7
40-2, hip		6
40-3, hip		1
40-4, hip		1
40-5, hip		2

*ARCO* association research circulation osseous.

A high resolution (pixel matrix, 512 × 512) helical CT scanner (CT High Speed Advantage; GE Medical Systems, Milwaukee, WI, USA) was used to obtain axial images from the anterior superior iliac spine to the knee joint through the distal femoral condyles. The slice thickness and interval were each set to 1 mm, and the table speed was set to 1 mm/s. ZedHip® (LEXI Co., Ltd., Tokyo, Japan) software was used for impingement simulation analysis. Digital imaging and communication in medicine (DICOM) data for each patient were transferred to ZedHip®, and three-dimensional (3D) simulation models of the pelvis and femur were constructed^19^. The software also simulates preoperative THA planning and ROM until impingement occurs between the implant and the bone, using the implant database provided by the implant manufacturer^20^.

**Figure 1:**
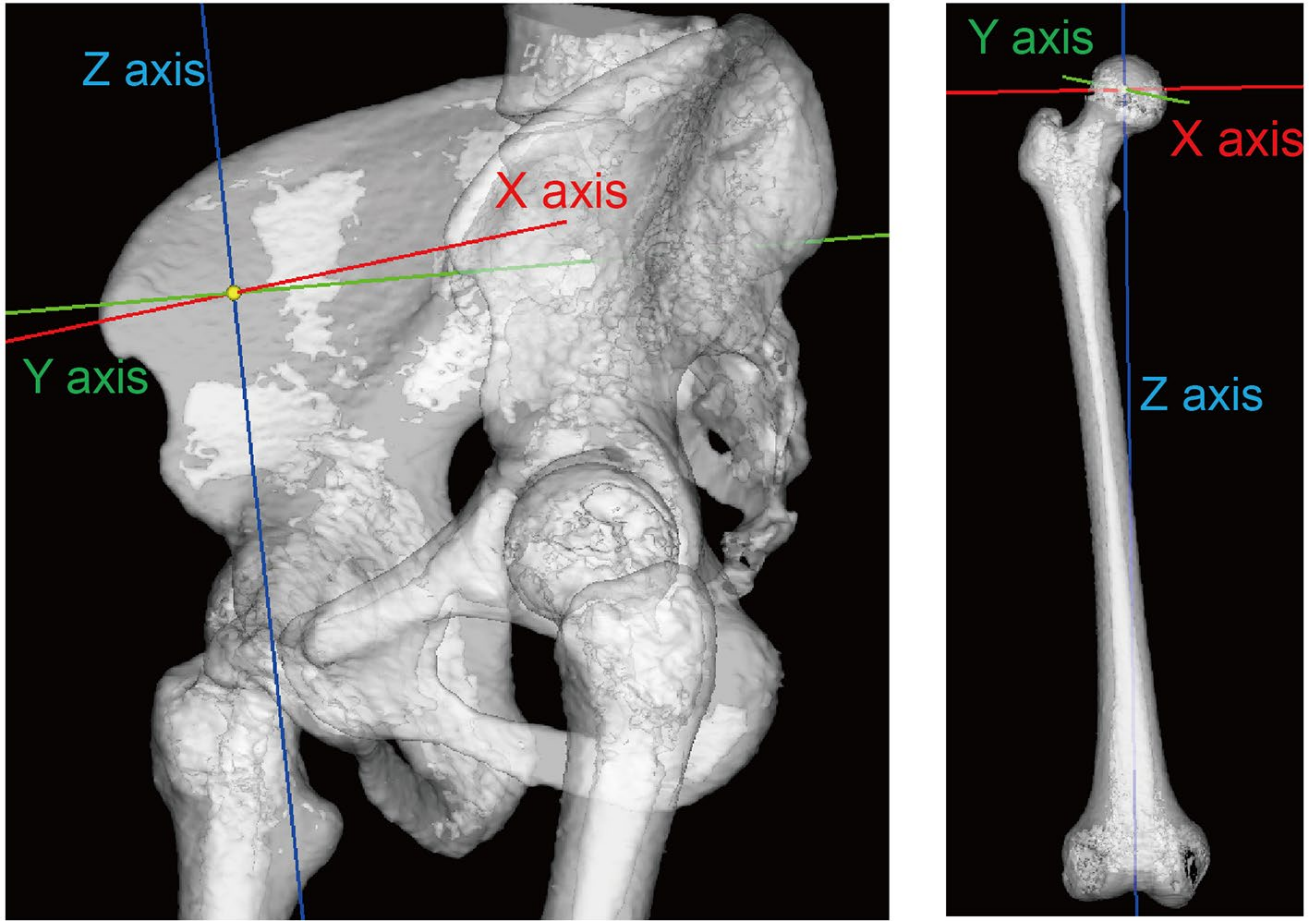
Left: The pelvis in the anterior pelvic plane coordinate system. Right: The right femur in the International Society of Biomechanics coordinate system.

**Figure 2:**
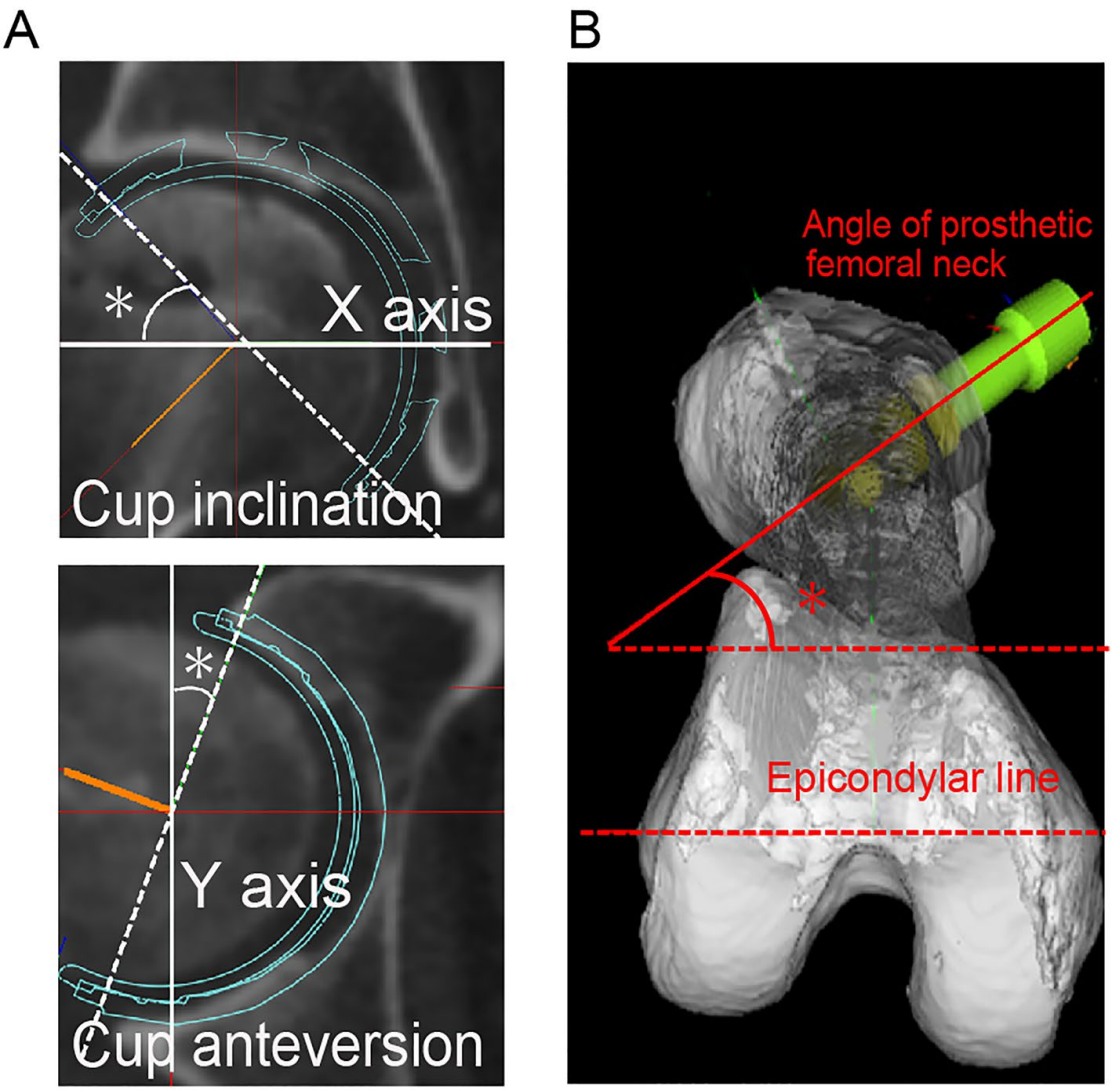
(**A**) The definitions of cup inclination (upper, white asterisk) and anteversion (lower, white asterisk) in the functional pelvic plate. (**B**) Stem anteversion was defined as the angle formed between the proximal femoral stem axis and the tangential line to the bilateral posterior femoral condylar margin on the tabletop plane. The red asterisk indicates the postoperative stem anteversion.

The pelvic coordinate system was defined based on the anatomical pelvic plane (APP): the X-axis was defined as the line connecting the right and left anterior superior iliac spines, Z-axis as the line passing through the midpoint of the anterior superior iliac spines and the pubic tubercle, and Y-axis as the line perpendicular to the X- and Z-axes^21^ (Fig. 1). The femoral coordinate system was defined based on the International Society of Biomechanics: the X-axis was defined as the line connecting both epicondyles of the knee, Z-axis as the line connecting the midpoints of both epicondyles of the knee and the center of the femoral head, and Y-axis as the line perpendicular to the X- and Z-axes^22^. Cup inclination and anteversion were defined as radiographic inclination and anteversion, respectively, as previously reported^23^ (Fig. 2A). Stem anteversion was defined as the angle of the prosthetic femoral neck relative to the epicondylar line^24^ (Fig. 2B).

### Computer simulation study

The femoral component was stimulated using the Exeter offset size 37.5 mm stem (Stryker, Newbury, UK) and VLIAN offset size 40 mm stem (Teijin Nakashima Medical, Okayama, Japan), a 125° neck angle on the prosthesis, and polished tapered cemented femoral components. For the stem implant size, we chose the largest size to accommodate a 2 mm cement mantle^25,26^. In this study, cup size varied from 48 to 56 mm, and stem size varied from 37.5-0 to 37.5-3 (Exeter) and 40-1 to 40-5 (VLIAN) (Table 1).

The stem was positioned with the anteversion increasing every 10° from 0° to 40°. To investigate the prosthetic and bony impingement distance, we chose the optimal size of the cup and stem for each case, and a head with a 32 mm diameter (+0, +4, +7 [VLIAN stem] and + 8 mm [Exeter stem] longer head length) and a 32 mm flat liner in all cases. The Trident cup (Stryker, Kalamazoo, Michigan, USA) and the GS cup (Teijin Nakashima Medical, Okayama, Japan) were used to simulate the acetabular component. For the acetabular implant size, we selected the largest acetabular cup to ensure adequate coverage, assuming a press-fit installation^27–29^. The cup was positioned at a 45° inclination and 15° anteversion angle following Biedermann’s recommendation^30^.

Furthermore, two simulation tests were conducted. First, we examined the medial femoral offset (MFO)^31,32^ and ROM using the +0-mm head, ensuring alignment with the contralateral leg length. Subsequently, the same parameters were investigated using the longer head. MFO and ROM were then investigated when longer heads were used while the leg length was adjusted to be similar to that of the contralateral side. The neutral position of the hip was defined as the position at which both the pelvic and femoral coordinate systems were aligned rotationally and the femoral and acetabular centers were aligned. All rotations were applied according to the femoral head of center^33^. ROM was assessed based on the maximum internal rotation angle at 60° and 90° flexion, external rotation at 10° extension, and maximum abduction and flexion without impingement. This measurement approach aligns with conditions examined in recent studies that utilized the THA Dynamic Planning Software^34,35^.

### Statistical analysis

One-way repeated measures analysis of variance with the Tukey test for post hoc comparisons was used to investigate the efficacy of stem anteversion and a longer head. All statistical analyses were performed using the IBM SPSS version 23 software (SPSS Inc., Chicago, IL, USA), and statistical significance was set at *P* < 0.05.

## Results

Table 2 shows the variations of MFO with stem anteversion when using a longer head. As stem anteversion increased, the MFO decreased (*P* < 0.001). No differences were present in the mean increase in MFO between longer heads with or without adjustment for leg length discrepancy. The mean leg lengths using the + 4-mm and + 8-mm heads of Exeter stem were 2.61 mm (2.43–2.75 mm) and 5.24 mm (4.88–5.52 mm), respectively. The mean leg lengths using the + 4-mm and + 7-mm heads of the VLIAN stem were 2.54 mm (2.37–2.69 mm) and 4.47 mm (4.17–4.70 mm), respectively.

**Table 2:** Variation of medial femoral offset with stem anteversion when using a longer head.

	Stem anteversion				
	0°	10°	20°	30°	40°
MF offset					
Exeter stem					
+ 4 mm head	3.22 (0.01)	3.08 (0.11)	2.90 (0.15)	2.66 (0.19)	2.36 (0.23)
+ 8 mm head	6.44 (0.01)	6.15 (0.21)	5.80 (0.30)	5.31 (0.39)	4.85 (0.69)
VLIAN stem					
+ 4 mm head	3.28 (0.01)	3.14 (0.11)	2.97 (0.16)	2.72 (0.19)	2.44 (0.25)
+ 7 mm head	5.74 (0.01)	5.49 (0.18)	5.22 (0.29)	4.76 (0.34)	4.26 (0.43)

Data shows mean (standard deviation) mm.

**Figure 3:**
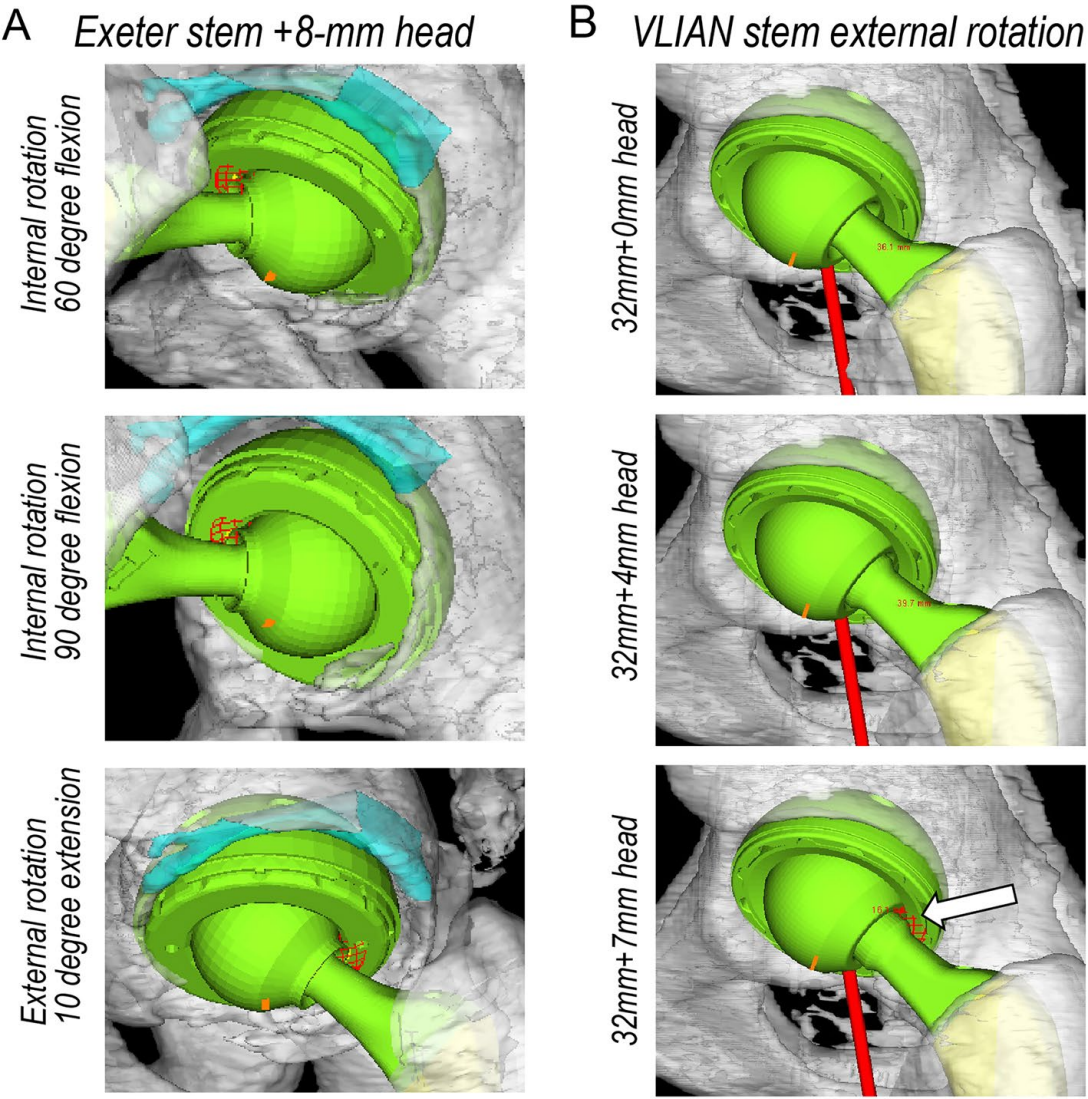
(**A**) The maximum internal rotation at 60° and 90° flexion and external rotation, respectively, at 10° extension of the + 8-mm head using the Exeter stem. (**B**) The maximum external rotation at 10° extension of each femoral head length using VLIAN stem. White arrow shows the impingement between liner and trunnion.

Table 3 (Exeter stem) and Table 4 (VLIAN stem) summarize the ROM without impingement during stem anteversion change and longer head use of each stem with or without adjustment for leg length discrepancy. As stem aversion increased, internal rotation (IR) at 60° and 90° flexion and maximum flexion increased (*P* < 0.001), while the external rotation (ER) at 10° extension decreased (*P* < 0.001). Increasing stem anteversion did not change the maximum abduction. In the Exeter stem, the IR (60° and 90° flexion), ER (10° extension), and maximum flexion increased using a + 4-mm longer femoral head. The IR (60° and 90° flexion), ER (10° extension), and maximum abduction decreased using an + 8-mm longer femoral head. These results were attributed to the observation that the skirt of the + 8-mm head caused prosthetic impingement (Fig. 3A). No differences were observed in the mean increase in ROM between longer heads with or without adjustment for leg length discrepancy (Table 3). In the VLIAN stem, although the IR (60° and 90° flexion) and maximum flexion increased using of a longer femoral head, no significant differences were found using the + 4-mm and + 7-mm heads (Table 4). The IR (60° and 90° flexion) did not significantly increase with stem anteversion. The ER (10° extension) of the + 4-mm head significantly increased compared with that of the 0-mm head. The ER (10° extension) and maximum abduction of the + 7-mm head decreased compared with those of the + 4-mm head. No differences were observed in the mean increase in ROM between longer heads with or without adjustment for leg length discrepancy (Table 4). These results were attributed to the observation that + 7-mm head induced exposure of the trunnion, causing prosthetic impingement in extension and external rotation and reducing ROM (Fig. 3B). We analyzed ROM with adjustments of a 5-degree increase or decrease in cup/liner inclination and anteversion in each instance and verified similar trend results.

**Table 3:** The mean range of motion between longer heads of Exeter stem with or without adjustment for leg length discrepancy.

	0 mm	+ 4-mm	+ 8-mm	+ 4-mm adjusted	+ 8-mm adjusted
Stem anteversion					
Internal rotation at flexion 60°					
0°	35.4	41.2	29.5	40.6	29.5
10°	47.2	51.2	39.9	51.4	39.9
20°	58.6	63.0	50.6	63.2	50.9
30°	68.9	73.1	61.2	73.2	61.4
40°	78.2	82.2	71.6	82.3	71.6
Internal rotation at flexion 90°					
0°	5.5	9.9	4.2	10.8	4.5
10°	16.9	21.2	12.7	22.5	12.9
20°	28.9	32.9	22.0	33.2	22.4
30°	40.1	43.8	31.6	44.2	31.8
40°	51.3	54.3	41.1	54.3	41.1
External rotation at extension 10°					
0°	59.5	62.5	45.9	62.5	45.9
10°	51.7	53.4	37.0	53.6	37.0
20°	42.5	44.2	27.6	44.3	27.7
30°	31.0	33.2	18.5	33.4	18.2
40°	17.0	19.8	9.1	19.9	9.1
Maximum abduction					
0°	58.2	58.2	49.6	58.2	49.7
10°	58.1	58.1	49.2	58.2	49.4
20°	58.1	58.1	48.6	58.1	48.6
30°	58.0	57.9	48.4	58.0	48.3
40°	58.0	57.9	47.6	57.9	47.7
Maximum flexion					
0°	108.2	115.4	114.2	115.4	114.2
10°	110.3	119.3	119.1	119.3	119.0
20°	120.5	125.2	125.4	125.1	125.3
30°	123.9	129.4	129.4	129.4	129.2
40°	124.6	133.2	132.7	133.1	132.5

Data show the mean, degree.

**Table 4:** The mean range of motion between longer heads of VLIAN stem with or without adjustment for leg length discrepancy.

	0 mm	+ 4 mm	+ 7 mm	+ 4 mm adjusted	+ 7 mm adjusted
Stem anteversion					
Internal rotation at flexion 60°					
0°	21.3	27.0	29.7	27.5	30.3
10°	36.7	42.8	44.5	42.2	43.9
20°	49.6	56.0	56.3	54.5	55.3
30°	60.5	67.1	68.0	66.1	66.9
40°	72.4	77.9	78.8	76.5	77.4
Internal rotation at flexion 90°					
0°	2.8	4.5	5.8	5.7	7.0
10°	8.8	13.1	14.0	19.3	15.5
20°	18.3	23.3	24.1	25.2	26.1
30°	31.4	37.2	37.0	38.7	38.8
40°	42.9	48.4	47.7	49.4	48.5
External rotation at extension 10°					
0°	61.2	63.1	57.8	63.1	57.8
10°	52.4	54.1	49.2	54.1	49.2
20°	42.8	44.8	40.8	44.4	40.8
30°	29.3	32.1	30.0	31.1	28.8
40°	16.3	20.7	19.8	19.2	18.2
Maximum abduction					
0°	61.2	61.3	59.5	61.3	59.5
10°	61.1	61.2	59.1	61.2	59.1
20°	61.1	61.1	58.7	61.1	58.7
30°	61.0	61.1	58.5	61.1	58.5
40°	61.0	61.0	58.2	61.0	58.2
Maximum flexion					
0°	106.1	115.7	114.6	115.3	113.2
10°	109.9	117.4	117.2	117.2	117.1
20°	116.2	124.7	124.2	124.4	123.9
30°	120.8	128.3	127.2	128.2	127.3
40°	122.9	131.5	130.2	131.0	130.9

Data show the mean, degree.

## Discussion

To the best of our knowledge, this study is the first to examine and elucidate the specific impacts of cemented stems and longer heads on offset, leg length, and impingement. Additionally, two types of cemented stems (Exeter and VLIAN stems) were investigated to validate the effects of different stem designs. This study demonstrated that a longer head increased the medial femoral offset along with the leg length and that these changes depended on stem anteversion. Additionally, a + 4-mm head increased ROM without impingement across both stem types, as demonstrated in Tables 3 and 4. Consequently, these results indicate that employing a head that is + 4-mm longer could be an intraoperative technique to improve dislocation resistance alongside adjustments to stem anteversion. Additionally, this simulation study revealed no differences in ROM using the longer head with or without adjustment for leg length discrepancy, suggesting that leg length extension is not directly related to ROM and that an increased offset may improve ROM without impingement. Since the cemented stem is less affected by the size and shape of the medullary cavity and the anteversion of the stem^36^ is relatively easy to adjust, intraoperative techniques and using a longer head can achieve a more suitable offset and achieve ROM increase without leg length discrepancy.

The finding that using a + 4-mm longer head can increase ROM without impingement is consistent with and attributable to recent 3D CT-based simulation studies that demonstrated that a high-offset stem can increase ROM without impingement^14^. In the present study, no significant increase in ROM was found using the + 7-mm and + 8-mm heads compared with the + 4-mm head. The decrease in external rotation and abduction in our simulation study could be attributed to the skirt (+ 8-mm head) and exposure of the trunnion (+ 7-mm head) (Fig. 2). Considering that skirted necks, larger trunnions, and smaller femoral heads have been reported to decrease ROM^37,38^, surgeons should carefully consider the stem design preoperatively. Additionally, a previous study reported that + 8-mm femoral heads exhibited greater fretting damage at the head–neck taper interface than all other head lengths^39^. Therefore, caution is recommended when using a longer head that exposes the trunnion.

Despite these findings, our study had several limitations. First, because this was a simulation study using a 3D CT bone model, the efficacy of soft tissue could not be assessed. However, several studies have explored the relationship between soft tissue and dislocation^40,41^. A cadaver study should be undertaken to further validate our research findings. Second, only two implants were used in this study, and similar trends were observed in the two designs examined. Future studies are required for further validation with different implant designs. Third, because this simulation study focused on the association between femoral head length, leg lengthening, and ROM, we selected and investigated hips that did not show DDH and OA changes. Therefore, future studies are required to confirm the impact on ROM without impingement using longer heads under pathological conditions.

## Conclusions

Enhancing dislocation resistance by solely increasing the offset with a longer head while simultaneously adjusting the depth of stem insertion may be a beneficial intraoperative technique. Although a + 4-mm longer head seems to bolster dislocation resistance, heads extended by + 7 or + 8 mm might not guarantee the same advantage. Therefore, surgeons should consider that certain implant designs, particularly with heads lengthened to + 7 or + 8 mm, might pose unintended complications.

## Data Availability

The dataset of this study is not publicly available. However, on reasonable request, derived data supporting the findings of this study are available from the corresponding author after approval from the Ethical Committee of the Hokkaido University Hospital.
