# Author Correction: Effect of longer femoral head on leg length, offset, and range of motion in total hip arthroplasty: a simulation study

**DOI:** 10.1038/s41598-024-59221-1

**Published:** 2024-04-11

**Authors:** Tomohiro Shimizu, Takuji Miyazaki, Shunichi Yokota, Hotaka Ishizu, Daisuke Takahashi, Norimasa Iwasaki

**Affiliations:** Department of Orthopaedic Surgery, Faculty of Medicine and Graduate School of Medicine, Hokkaido University, Kita-15, Nish-7, Kita-ku, Sapporo, 060-8638 Japan

Correction to: *Scientific Reports* 10.1038/s41598-024-52264-4, published online 21 January 2024

The Competing interest section in the original version of this Article was incomplete. The original version of the statement, which read

“The authors declare no competing interests.”

now reads

“Dr. Takahashi’s work has been funded by Teijin Nakashima Medical and Stryker Japan K.K. Dr. Shimizu, Dr. Miyazaki, Dr. Ishizu, Dr. Yokota and Dr. Iwasaki declare no potential conflict of interest.”

The original Article has been corrected.

